# SB‐216763, a GSK‐3β inhibitor, protects against aldosterone‐induced cardiac, and renal injury by activating autophagy

**DOI:** 10.1002/jcb.26788

**Published:** 2018-03-30

**Authors:** Yi‐De Zhang, Xiao‐Jun Ding, Hou‐Yong Dai, Wei‐Sheng Peng, Nai‐Feng Guo, Yuan Zhang, Qiao‐Ling Zhou, Xiao‐Lan Chen

**Affiliations:** ^1^ Department of Nephrology Affiliated Hospital of Nantong University Nantong Jiangsu China; ^2^ Department of Cardiology Affiliated Danyang People's Hospital of Nantong University Danyang China; ^3^ Department of Nephrology Affiliated Xiangya Hospital of Central South University Changsha Hunan China

**Keywords:** aldosterone, autophagy, cardiac and renal injury, GSK‐3β inhibitor

## Abstract

Cardiovascular and renal inflammation induced by Aldosterone (Aldo) plays a pivotal role in the pathogenesis of hypertension and renal fibrosis. GSK‐3β contributes to inflammatory cardiovascular and renal diseases, but its role in Aldo‐induced hypertension, and renal damage is not clear. In the present study, rats were treated with Aldo combined with SB‐216763 (a GSK‐3β inhibitor) for 4 weeks. Hemodynamic, cardiac, and renal parameters were assayed at the indicated time. Here we found that rats treated with Aldo presented cardiac and renal hypertrophy and dysfunction. Cardiac and renal expression levels of molecular markers attesting inflammation and fibrosis were increased by Aldo infusion, whereas the treatment of SB‐216763 reversed these alterations. SB‐216763 suppressed cardiac and renal inflammatory cytokines levels (TNF‐a, IL‐1β, and MCP‐1). Meanwhile, SB‐216763 increased the protein levels of LC3‐II in the cardiorenal tissues as well as p62 degradation, indicating that SB‐216763 induced autophagy activation in cardiac, and renal tissues. Importantly, inhibition of autophagy by 3‐MA attenuated the role of SB‐216763 in inhibiting perivascular fibrosis, and tubulointerstitial injury. These data suggest that SB‐216763 protected against Aldo‐induced cardiac and renal injury by activating autophagy, and might be a therapeutic option for salt‐sensitive hypertension and renal fibrosis.

**Abbreviations**
Aldo: Aldosterone
Col I: Collagen type I
TGF‐β: Transforming growth factor‐β
TNF‐α: Tumor necrosis factor α

## 1 INTRODUCTION

Aldosterone (Aldo) secreted from the adrenal cortex plays a crucial role in regulating renal sodium transport and electrolytic balance through the activation of mineralocorticoid receptor (MR) in the kidney.^1^, ^2^ Clinical studies demonstrated that inhibition of MR could decrease the risk of both morbidity and mortality in patients with heart failure, and inhibit albumin excretion in hypertensive and diabetic patients.^3^, ^4^, ^5^ In addition, MR antagonists also present a renoprotective effect in several experimental models of kidney disease.^6^, ^7^

Aldo is implicated in cardiovascular and renal remodeling by inducing inflammation, oxidative stress, fibrosis, and hypertrophy.^2^, ^8^, ^9^ Previous studies showed that chronic inflammation has a critical role in the pathogenesis of hypertension,^10^, ^11^ and renal inflammation is correlated with the development and progression of renal damage.^12^, ^13^ These findings suggest that Aldo‐induced inflammation might be used as a potential therapeutic target for treating salt‐sensitive hypertension and renal fibrosis.^14^

Glycogen synthase kinase 3β (GSK3β) is a multifunctional serine/threonine kinase. GSK3β is involved in the growth of the heart during development and in response to stress.^15^ However, its role in regulating cardiac and renal injury remains unclear. GSK3β has a broad range of substrates, and regulates inflammatory response, cell differentiation, and survival.^16^, ^17^ GSK3β is an important positive regulator of inflammatory process.^18^, ^19^, ^20^, ^21^ GSK3β‐deficient cells become more sensitive to tumor necrosis factor α (TNF‐α)‐induced apoptosis.^22^ Recent studies demonstrated that the therapeutic effect of GSK3β inhibitor is associated with suppressing inflammatory response. Inhibition of GSK3β results in decreased activation of the pro‐inflammatory transcription factor NF‐κB. Additionally, GSK3β inhibition contribute to produce anti‐inflammatory cytokine IL‐10.^23^

GSK3β inhibition triggers a profound autophagic response in cells under serum‐free condition.^24^ This phenomenon was also observed in vivo from ischemic mouse models.^25^, ^26^ However, the mechanism underlying GSK3β inhibition‐triggered autophagy is not fully clear. Autophagy is a lysosome‐mediated intracellular catabolic process by which cells remove their damaged organelles for the maintenance of cellular homeostasis.^27^ Autophagy is induced in response to intracellular or extracellular signals, such as starvation, pathogen infection, and endoplasmic reticulum stress.^28^, ^29^ Emerging evidence has indicated that autophagy may have an essential role for the host during bacterial clearance and may also interact with inflammatory processes, which consequently may impact the outcomes of disease progression.^30^, ^31^

Based on the above findings, we investigated whether GSK3β inhibition protects against Aldo‐induced cardiac and renal injury by activating autophagy. Current data suggest that rats treated with Aldo present cardiac and renal injury. The treatment of SB‐216763 reverses these alterations. SB‐216763 suppressed cardiovascular and renal inflammation by activating autophagy in cardiac and renal tissues.

## 2 MATERIALS AND METHODS

### 2.1 Animal models

The study was approved by the Ethics Committee of Nantong University. Adult male Wistar rats were obtained from the Chinese Academy of Sciences (Shanghai, China), and maintained in a pathogen‐free facility. The animals were divided into four groups (*n* = 9/group): (1) Vehicle infusion group treated with vehicle alone; (2) Aldo‐salt group treated with an infusion of Aldo‐salt (1 mg/kg/day diluted in sunflower oil and administered by subcutaneous injection); (3) Aldo‐salt plus SB‐216763 group treated with an infusion of Aldo‐salt plus SB‐216763 at 1.5 mg/kg/day (MCE, Princeton, NJ); and (4) SB‐216763 group. This dose was chosen on the basis of previous studies reporting its anti‐inflammatory role.^32^, ^33^ After 4 weeks of treatment, urine was collected in metabolic cages, and hemodynamic parameters were assayed.^34^ For example, blood pressure (BP) was measured in conscious but restrained animals, prewarmed to 34°C for 20 min. For each group, BP was measured three times on 3 separate days, and the mean value of all readings was taken as the average for the rat. Then blood samples, heart and kidney tissues were collected under sedation with sodium pentobarbital anesthesia.

### 2.2 Quantitative real‐time PCR (qPCR)

Total RNA was extracted from rat heart or kidney using Trizol reagent (Invitrogen, Carlsbad, CA). The reverse transcription (RT) was carried out using Oligo dT primer (Takara, Osaka, Japan). qPCR was performed using a standard protocol from the SYBR Green PCR kit (Toyobo, Osaka, Japan) on Applied Biosystems 7300 real‐time PCR system. β‐actin was used as internal control. The qPCR primers are as follows:

TNF‐a forward, CTTCTGTCTACTGAACTTCGGG,

TNF‐a reverse, GTTGTCTTTGAGATCCATGCC;

MCP‐1 forward, GCTGACCCCAATAAGGAATG,

MCP‐1 reverse, CTTGAGGTGGTTGTGGAAAAGA;

IL‐1beta forward, ATGATGACGACCTGCTAGTGTGT,

IL‐1beta reverse, TGGCTTATGTTCTGTCCATTGAG;

TGF‐beta forward, CAACGCAATCTATGACAAAACC,

TGF‐beta reverse, ACAAGAGCAGTGAGCACTGAAG;

Col1 forward, TCCTTCTGGTCCTCGTGGTCTCC,

Col1 reverse, TTCCCCATCATCTCCGTTCTTGC.

### 2.3 Western blot and antibodies

Western blot analysis to assess rat LC3‐I, LC3‐II, p62, and β‐actin protein expression was performed as previously described.^35^ The anti‐ LC3‐I/LC3‐II/p62 primary antibodies were purchased from Santa Cruz Biotechnology (Santa Cruz, CA). β‐actin primary antibodies were purchased from Sigma (St. Louis, MO). Briefly, equivalent amounts of total protein (150 μg) were electrophoresed through a 12% SDS‐polyacrylamide gel, and then wet electro‐transferred to 0.2 μm PVDF membranes (Bio‐Rad, Richmond, CA). The blots were incubated at 37°C for 2 h with indicated antibodies, and then incubated with a goat anti‐rabbit HRP‐conjugated secondary antibody (1:5000, Jackson, Bar Harbor, ME). Protein signals were visualized by enhanced chemiluminescence detection (Pierce Biotechonology, Rockford, IL). The intensity of the selected bands was quantified using Image J.

### 2.4 Enzyme‐linked immunosorbent assay (ELISA)

The hearts and kidneys were removed and homogenized at the indicated time. Homogenates were sonicated for 30 s and then centrifuged at 2500*g* and 4°C. The supernatants were used for measurement of TNF‐a, MCP‐1, and IL‐1β. ELISAs were performed using a TNF‐a kit (R&D Systems, Minneapolis, MN), MCP‐1 kit (R&D Systems), and IL‐1β kit (R&D Systems) according to the manufacturers’ protocols.

### 2.5 Histological analysis

Rats were treated with Aldo or SB‐216763 for 4 weeks, and kidney tissues or left ventricles were quickly fixed with buffered 4% paraformaldehyde, embedded in paraffin and cut into 4 µm‐thick sections. Periodic acid‐Schiff and Masson's trichrome staining were performed using serial sections. Tubulointerstitial fibrosis areas were semiquantified using image J software and expressed as a percentage of the total area. Perivascular fibrosis was assessed by calculating the percentage of Trichrome‐stained collagen deposits surrounding the vessel to the total perivascular area using the software's color cube function.

### 2.6 Fluorescence microscopy analysis

Transverse sections at 3 µm thickness were fixed with 3% paraformaldehyde, and subjected to immunocytochemistry as previously described.^32^, ^33^ The sections were briefly rinsed in PBS and blocked in a solution containing 5% BSA (Sigma) and 0.1% Triton X‐100 (Sigma) for 1 h at room temperature. The slides were then immunostained with primary antibodies against LC3β (3868#, Cell Signaling Technology, Danvers, MA). To visualize the primary antibodies, slides were stained with FITC‐conjugated secondary antibodies. The slides were also stained with 40, 6‐diamidino‐2‐phenylindole (DAPI) to visualize the nuclei. After washed thrice in PBS samples were examined under a fluorescence laser scanning confocal FV1000 microscope (Olympus).

### 2.7 Statistical analysis

All data are expressed as mean ± SEM, computed from the average measurements obtained from each group of animals. Results were analysed using unpaired Student's *t*‐test or the Mann‐Whitney U‐test. Analyses were conducted using Graph­Pad Prism (4.0) (software, Inc. San Diego, CA). Differences were deemed statistically significant at *P* < 0.05.

## 3 RESULTS

### 3.1 SB‐216763 suppresses aldo‐induced cardiorenal inflammation

**Figure 1:**
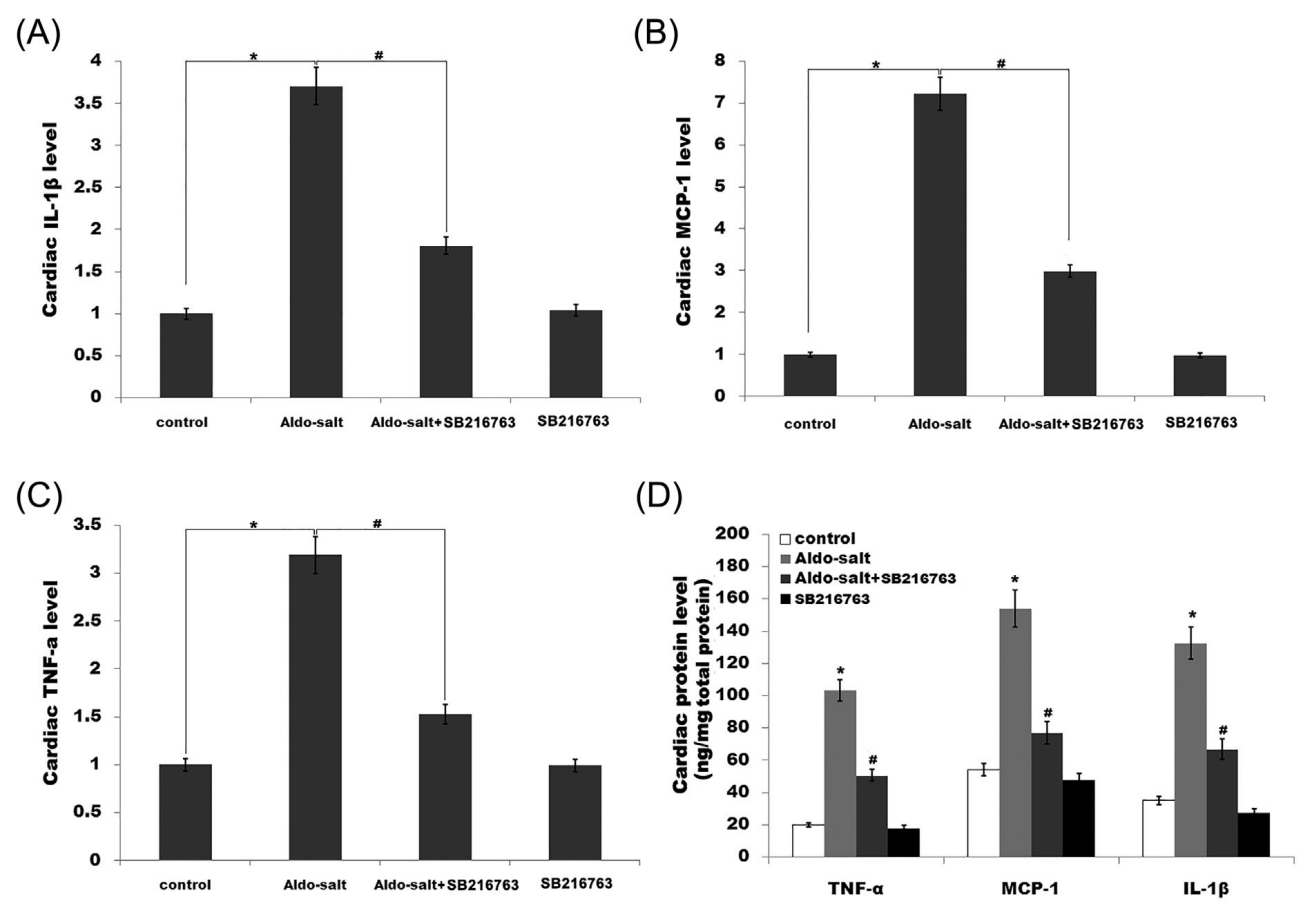
SB‐216763 suppresses cardiac inflammation and fibrosis caused by Aldo‐salt. Cardiac (A) and renal (B) protein expression of total GSK3β and p‐GSK3β were assayed in cardiorenal tissues treated with Aldo‐salt or with Aldo‐salt plus SB‐216763. *P < 0.05 versus control, ^#^ *P* < 0.05 versus Aldo‐salt. (C‐E) Cardiac mRNA levels of TNF‐α, MCP‐1, and IL‐1β in rats treated with Aldo‐salt or with Aldo‐salt plus SB‐216763. *P < 0.05 versus control, ^#^ *P* < 0.05 versus Aldo‐salt. (F) Cardiac protein levels of TNF‐α, MCP‐1, and IL‐1β in rats treated with Aldo‐salt or with Aldo‐salt plus SB‐216763 were assayed using ELISA. *P < 0.05 versus control, ^#^ *P* < 0.05 versus Aldo‐salt

Aldo‐treated rats present a significant increase in systolic blood pressure (SBP) and diastolic BP (DBP) (Table 1). Meanwhile, Aldo treatment results in an increase in ratio of heart weight to body weight and a decrease in heart rate (Table 1). Both cardiac dysfunction and hypertrophy are reversed by SB‐216763, a GSK‐3β inhibitor (Table 1). In addition, body weight, urine volume, serum creatinine, creatinine clearance, kidney weight/body weight ratio, and urinary protein excretion of each group at the end of the 4‐week experiment are present in Table 2. Aldo‐treated rats induce renal hypertrophy, increase glomerular filtration rate, and results in a significant increase in serum creatinine compared with the other groups. Both renal dysfunction and hypertrophy are prevented by SB‐216763 treatment. We assayed the effect of SB‐216763 on regulating GSK3β expression and activation. As shown in Figures 1A and 1B, SB‐216763 increases phosphor‐GSK‐3βS9 (PSer9) levels, but not total GSK‐3β levels in the heart and kidney.

**Table 1:** Physiological and hematological parameters in Aldo‐treated rats

	Control	Aldo	Aldo + SB216763
SBP, mm Hg	132 ± 0.8	151 ± 0.5^*^	138 ± 0.9^**^
DBP, mm Hg	94 ± 1.1	115 ± 0.7^*^	104 ± 1.3^**^
HR, beats/min	327 ± 4.3	277 ± 7.2^*^	311 ± 6.4^**^
HW/BW, mg/g	2.56 ± 0.03	2.88 ± 0.01^*^	2.67 ± 0.01^**^

Aldo, aldosterone; SBP, systolic blood pressure; DBP, diastolic blood pressure; HW, heart weight; BW, body weight.

Values are presented as mean ± SEM.

^*^ *P* < 0.05 versus control.

^**^ *P* < 0.05 versus Aldo group.

**Table 2:** Physiological and renal parameters in Aldo‐treated rats

	Control	Aldo	Aldo + SB216763
Creatinine clearance, mL/min	1.21 ± 0.16	1.34 ± 0.52	1.19 ± 0.66
Urine volume (mL/day)	11.5 ± 2.3	36.2 ± 5.7^*^	24.6 ± 5.9^**^
Serum creatinine (mg/dL)	0.74 ± 0.19	1.36 ± 0.32^*^	1.06 ± 0.42^**^
KW/BW, mg/g	2.81 ± 0.01	3.93 ± 0.02^*^	3.01 ± 0.01^**^

Aldo, aldosterone; KW, kidney weight; BW, body weight.

Values are presented as mean ± SEM.

^*^ *P* < 0.05 versus control.

^**^ *P* < 0.05 versus Aldo group.

**Figure 2:**
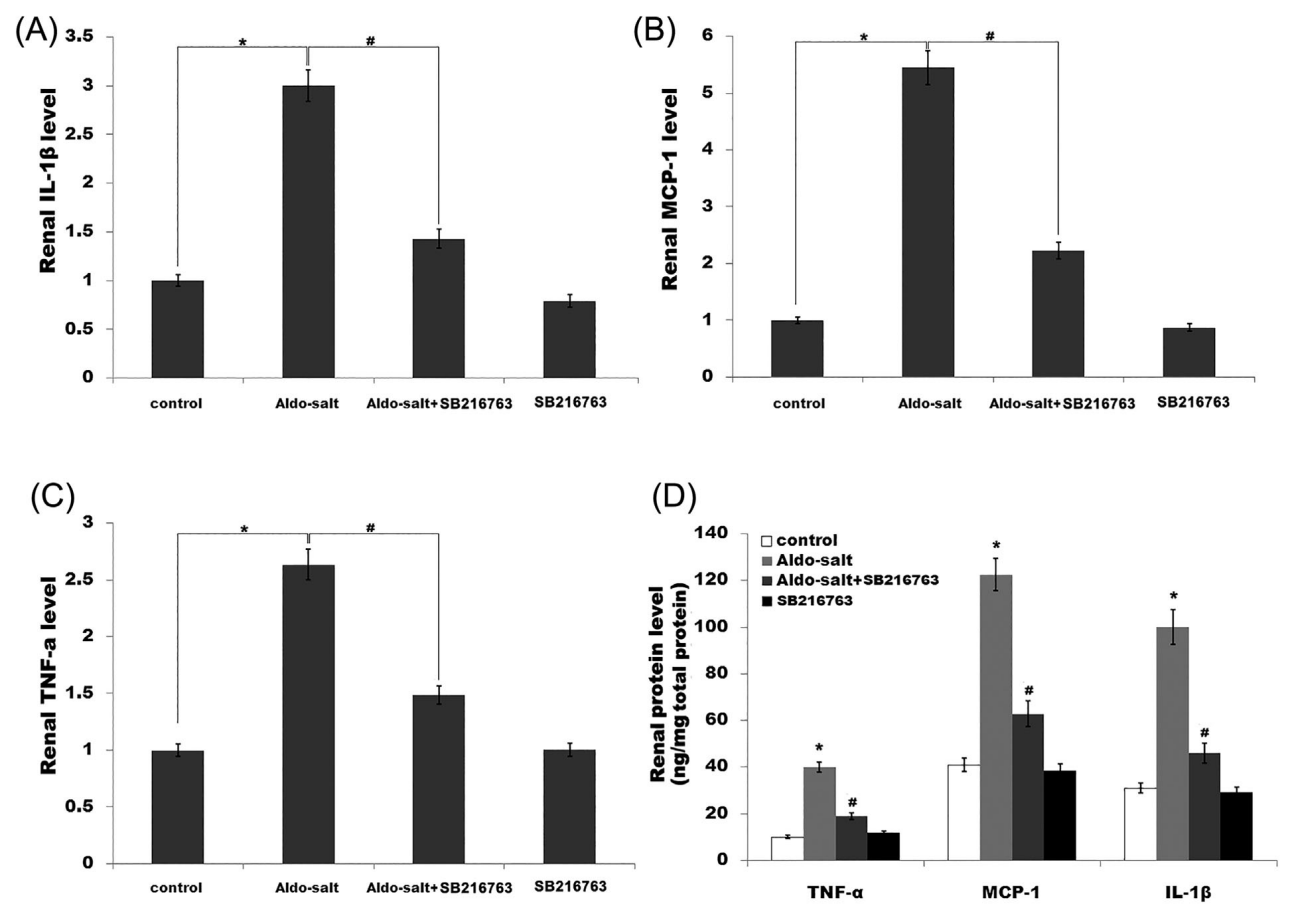
SB‐216763 suppresses renal inflammation and fibrosis caused by Aldo‐salt in vivo. (A‐C) Renal mRNA levels of TNF‐α, MCP‐1, and IL‐1β in rats treated with Aldo‐salt or with Aldo‐salt plus SB‐216763. *P < 0.05 versus control, ^#^ *P* < 0.05 versus Aldo‐salt. (D) Renal protein levels of TNF‐α, MCP‐1, and IL‐1β in rats treated with Aldo‐salt or with Aldo‐salt plus SB‐216763 were assayed using ELISA. *P < 0.05 versus control, ^#^ *P* < 0.05 versus Aldo‐salt

Previous studies demonstrated that inflammation plays an important role in the pathogenesis of hypertension, and the development and progression of renal fibrosis.^13^, ^36^, ^37^ We then assayed the expression of various proinflammatory cytokines by qPCR and ELISA. Figure 1C‐F showed that the cardiac mRNA and protein levels of TNF‐a, MCP‐1, and IL‐1β are markedly increased by Aldo infusion, whereas SB‐216763 treatment inhibits cardiac inflammatory cytokines levels (Figure 1C‐F). Similarly, the renal inflammatory cytokines levels (TNF‐a, MCP‐1, and IL‐1β) are enhanced after Aldo treatment, whereas the expression of these genes is inhibited by SB‐216763 (Figure 2A‐D).

### 3.2 SB‐216763 suppresses aldo‐induced cardiorenal fibrosis

**Figure 3:**
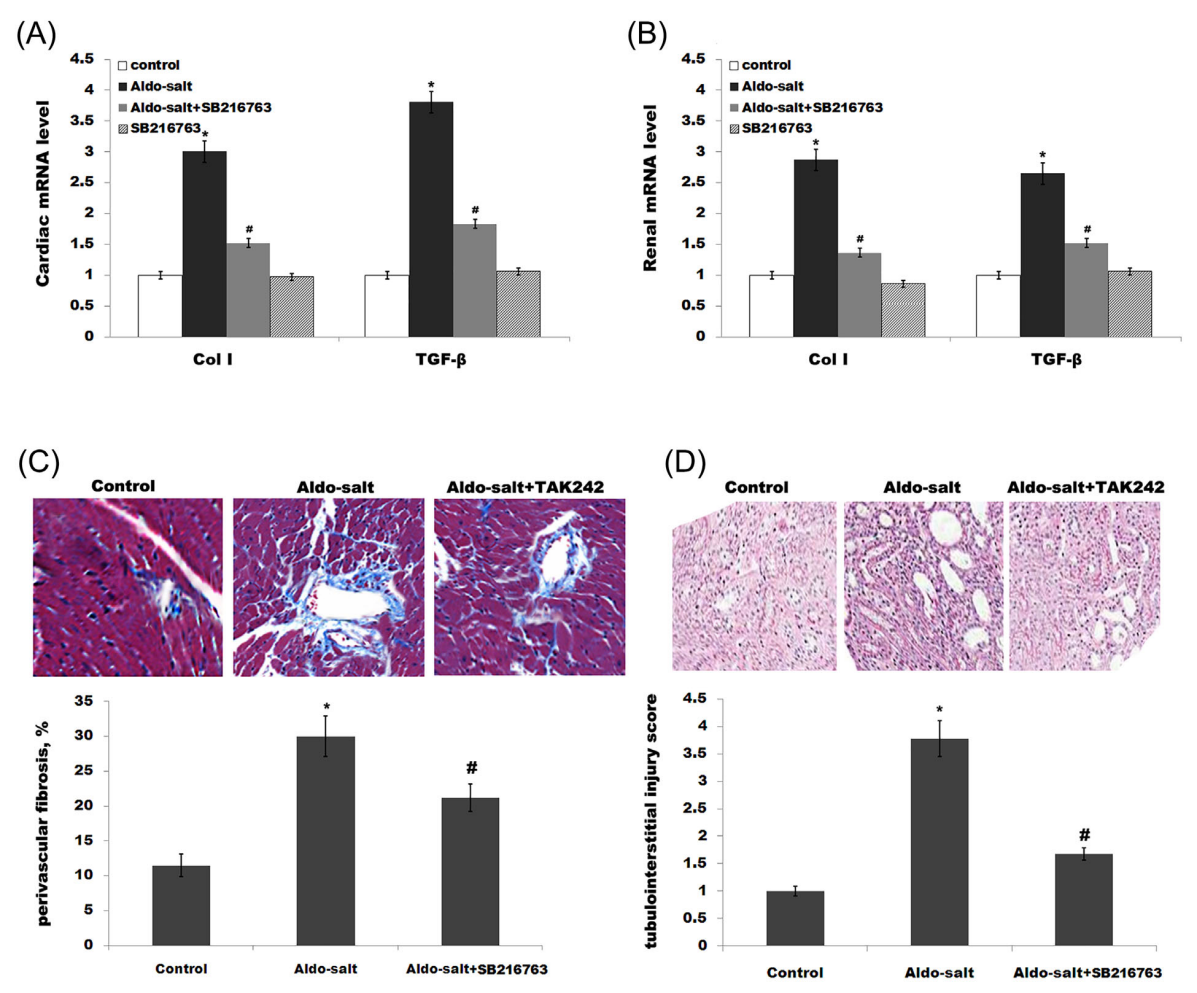
SB‐216763 suppresses Aldo‐induced cardiorenal fibrosis. Cardiac (A) or renal (B) mRNA levels of Col I and TGF‐β in rats treated with Aldo‐salt or with Aldo‐salt plus SB‐216763. *P < 0.05 versus control, ^#^ *P* < 0.05 versus Aldo‐salt. (C) Representative images and quantitation of perivascular fibrosis in left ventricles of rats treated with Aldo‐salt or with Aldo‐salt plus SB‐216763. *P < 0.05 versus control, ^#^ *P* < 0.05 versus Aldo‐salt. (D) Histological staining with periodic acid Schiff (PAS), also showing tubulointerstitial damage of rats treated with Aldo‐salt or with Aldo‐salt plus SB‐216763. *P < 0.05 versus control, ^#^ *P* < 0.05 versus Aldo‐salt. *n* = 9, 200 × magnification

To evaluate cardiorenal fibrosis, we assayed the expression of collagen type I (Col I) and transforming growth factor‐β (TGF‐β), which are extracellular matrix protein and profibrotic marker, respectively. Although the Aldo‐treated group shows an increase of Col I and TGF‐β in rat heart, the expression Col I and TGF‐β is inhibited by SB‐216763 treatment (Figure 3A). As in the case of heart, Aldo treatment upregulates the expression of renal Col I and TGF‐β, and SB‐216763 suppresses this increase (Figure 3B). Perivascular fibrosis in the left ventricle was assessed by deposition of collagen around the vasculature. Figure 3C presents representative images of collagen deposition and quantitation of fibrosis. SB‐216763 treatment markedly suppresses Aldo‐induced perivascular fibrosis. Meanwhile, periodic acid Schiff‐stained sections revealed that SB‐216763 inhibits Aldo‐induced tubulointerstitial damage (Figure 3D). These results suggest that SB‐216763 treatment inhibits cardiorenal inflammation and fibrosis induced by Aldo.

### 3.3 SB‐216763 increases autophagy activation in cardiorenal tissues

**Figure 4:**
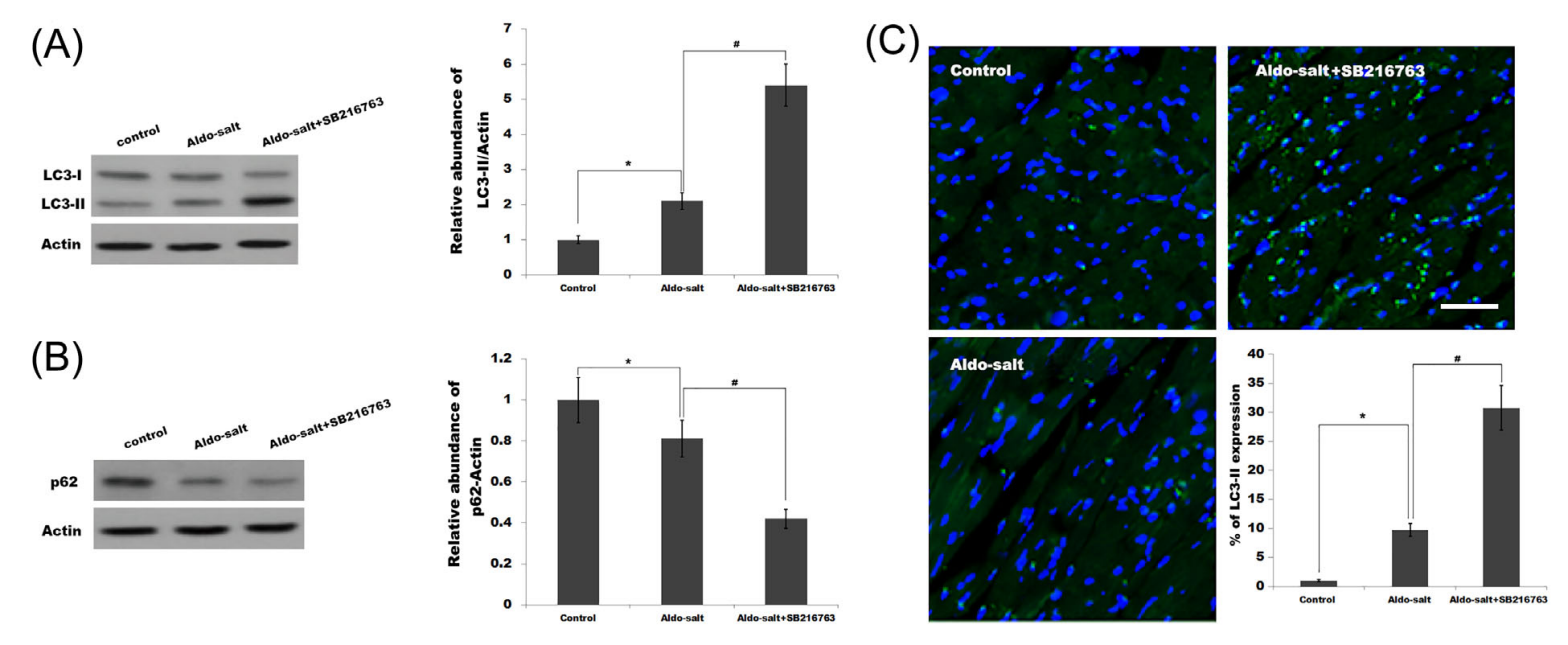
SB‐216763 activates cardiac autophagy. Cardiac protein expression of LC3‐I, LC3‐II (A), and p62 (B) were assayed in cardiac tissues treated with Aldo‐salt or with Aldo‐salt plus SB‐216763. *P < 0.05 versus control, ^#^ *P* < 0.05 versus Aldo‐salt. Representative micrographs (C) showing immunofluorescent staining for LC3‐II for among different groups as indicated. *P < 0.05 versus control, ^#^ *P* < 0.05 versus Aldo‐salt

**Figure 5:**
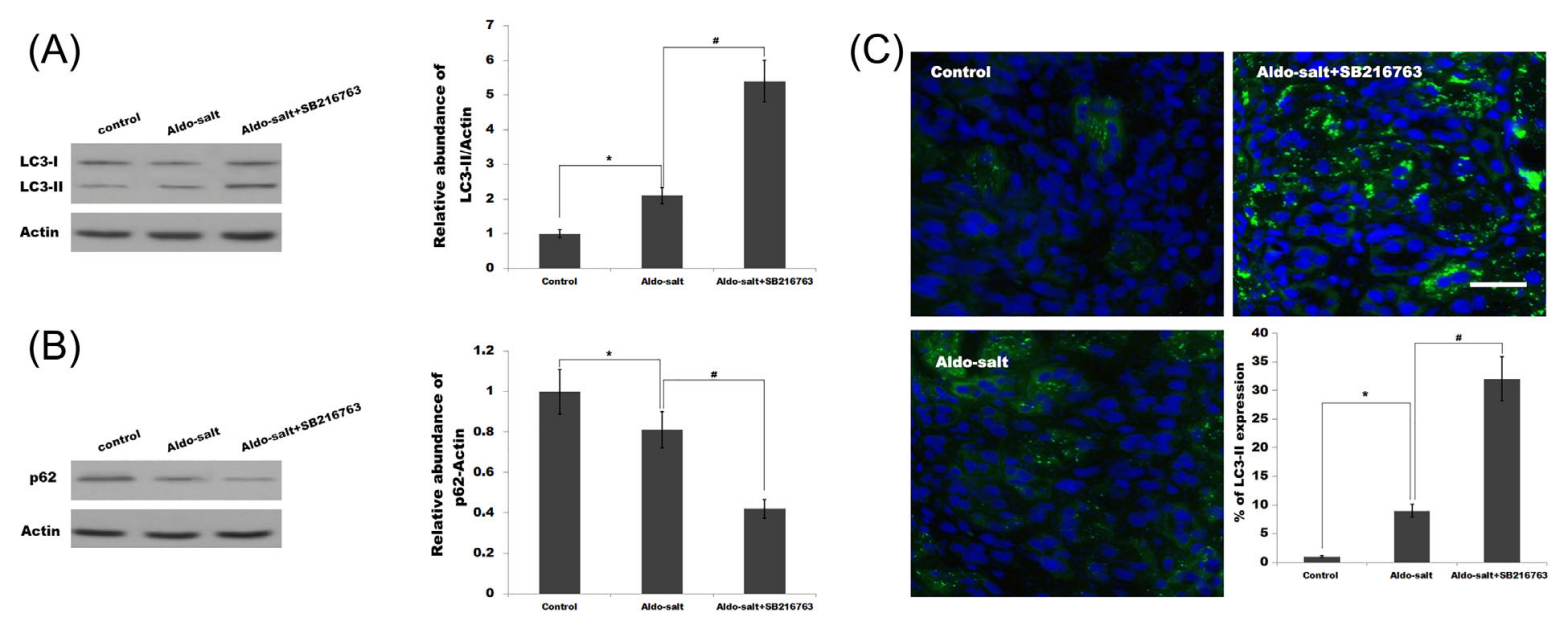
SB‐216763 activates renal autophagy. Renal protein expression of LC3‐I, LC3‐II (A) and p62 (B) were assayed in renal tissues treated with Aldo‐salt or with Aldo‐salt plus SB‐216763. SB‐216763 increased the Aldo‐salt‐induced autophagy. *P < 0.05 versus control, ^#^ *P* < 0.05 versus Aldo‐salt. Representative micrographs (C) showing immunofluorescent staining for LC3‐II for among different groups as indicated. *P < 0.05 versus control, ^#^ *P* < 0.05 versus Aldo‐salt

Recent studies demonstrated that GSK3β functions as a key regulator coordinating cellular homeostasis by suppressing autophagy in physiological and pathological processes such as cancer,^38^ axonal degeneration,^39^ and diabetes.^40^ Moreover, autophagy plays a crucial role in inflammation and fibrosis. Saitoh et al^41^ demonstrated that loss of the autophagy protein Atg16L1 enhances endotoxin‐induced IL‐1β production. Knockdown of autophagy increases the immune response in hepatitis C virus‐infected hepatocytes.^42^ The p62 protein, also called sequestosome 1 (SQSTM1), binds directly to LC3 and GABARAP family proteins via a specific sequence motif. The protein is itself degraded by autophagy and may be used as a marker to study autophagic flux.^43^ Here we found that the Aldo treatment slightly increases the protein levels of LC3‐II (the marker of autophagy activation) in the cardiorenal tissues as well as p62 degradation, whereas SB‐216763 treatment results in marked activation of autophagy (Figures 4A and 4B and 5A and 5B). The fluorescence analysis was carried out to further verify the activation of autophagy after Aldo or Aldo plus SB‐216763 treatment. Figure 4C and Figure 5C showed that following SB‐216763 treatment, there is a significant increase of LC3 green puncta representing autophagic vacuoles and an accumulation of LC3‐II in cardiorenal tissues, indicating that autophagy is activated.

### 3.4 SB‐216763 suppresses aldo‐induced cardiorenal injury by regulating autophagy

**Figure 6:**
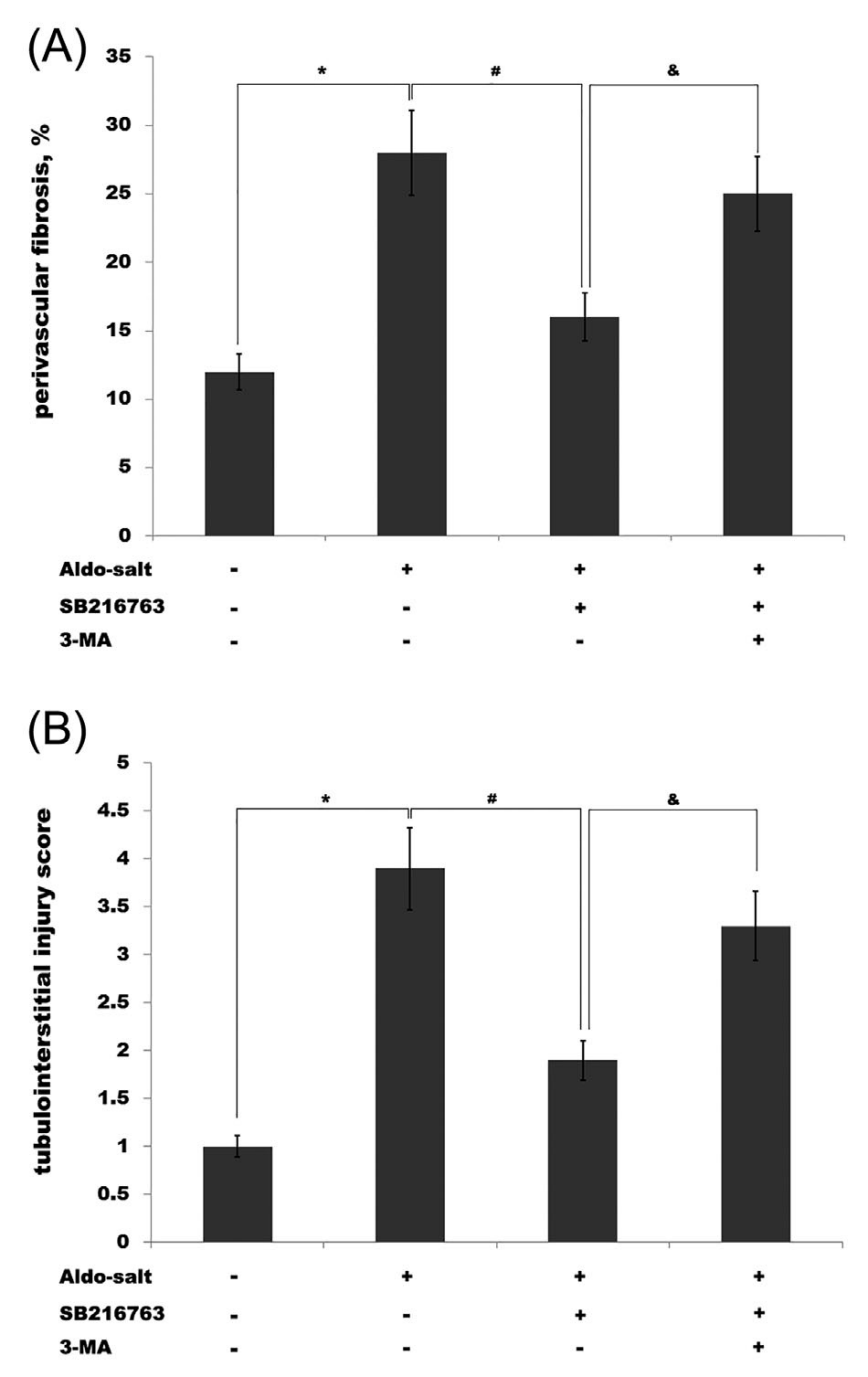
SB‐216763 suppresses Aldo‐induced cardiorenal injury by regulating autophagy. Rats were treated with Aldo (1 mg/kg/day), SB‐216763 (1.5 mg/kg/day) or 3‐MA (800 nmol) for 4 weeks, and the levels of perivascular fibrosis and tubulointerstitial injury were assessed using Periodic acid‐Schiff and Masson's trichrome staining. Quantitation of perivascular fibrosis in left ventricles (A) or tubulointerstitial damage in kidney tissues (B) was shown. ^*#&^ *P* < 0.05. *n* = 9. Inhibition of autophagy by 3‐MA partially destroys the role of SB‐216763 in inhibiting Aldo‐induced cardiorenal injury

SB‐216763 suppresses Aldo‐induced cardiorenal injury and activates autophagy, and autophagy plays important role in inhibiting proinflammatory response. Therefore, we next investigated whether SB‐216763 suppresses Aldo‐induced cardiorenal injury by activating autophagy. A 3‐methyladenine (3‐MA), which inhibits the formation of autophagosomes, attenuates LC3‐II upregulation. A 3‐MA is usually used to inhibit and study the mechanism of autophagy.^44^ As shown in Figures 6A and 6B, the level of perivascular fibrosis and tubulointerstitial injury score increases after Aldo treatment, whereas SB‐216763 can reverse the alteration. More important, pharmacological inhibition of autophagy by 3‐MA markedly inhibits the role of SB‐216763 in suppressing perivascular fibrosis and tubulointerstitial injury. These results demonstrate that SB‐216763, a GSK‐3β inhibitor, protects against Aldo‐induced cardiac, and renal injury by activating autophagy.

## 4 DISCUSSION

In this study, we investigated the potential role of SB‐216763, a GSK3β inhibitor, in treating salt‐sensitive hypertension, and renal fibrosis. The current data demonstrate that: (i) SB‐216763 inhibits Aldo‐induced cardiorenal inflammation; (ii) SB‐216763 inhibits Aldo‐induced cardiorenal fibrosis; (iii) SB‐216763 activates cardiorenal autophagy induced by Aldo; and (iv) SB‐216763 inhibits cardiorenal injury by activating autophagy. These results reveal the important role of SB‐216763 and autophagy in regulating salt‐sensitive hypertension and renal fibrosis, and suggest SB‐216763 might be used as a potential therapeutic target for treating salt‐sensitive cardiorenal injury.

Renal and cardiovascular fibrosis is found to be linked to inflammation in Aldo‐treated models.^45^ Inhibition of inflammatory cytokines ameliorates renal and cardiac injury in several experimental models.^46^ Here we demonstrate that Aldo treatment induces the production of pro‐inflammatory cytokines (such as TNF‐a, IL‐1β, and MCP‐1), whereas SB‐216763 markedly reverses these alterations in kidney and heart.

Autophagy is generally considered to be a cell survival mechanism that functions in response to various stress conditions and plays a critical role in human physiology and diseases, especially in inflammation and immunity.^47^, ^48^ Autophagy or autophagy‐related proteins could control inflammatory signaling by regulating inflammatory transcriptional responses. For example, upregulated p62 in autophagy‐deficient cells activate the pro‐inflammatory transcription factor NF‐κB.^49^ Autophagy also prevents tissue inflammation due to its role in apoptotic corpse clearance.^30^ Recent studies reported that impaired or deficient autophagy is believed to contribute to renal and cardiovascular disease as described in previous studies that focused on the role of autophagy in cardiac‐renal disease,^50^, ^51^ but the mechanism is not understood clearly. GSK3β can regulate autophagy, but its underlying mechanism remains unclear. Moreover, the different studies have given a different conclusion about the role of GSK3β in regulating autophagy. Sarkar et al^52^ reported that lithium (an inhibitor of GSK3β activity) can induce autophagy by inhibiting inositol monophosphatase, but another study showed that lithium can reduce autophagy and apoptosis after neonatal hypoxia‐ischemia.^53^ Inhibition of GSK3β activity using SB‐216763 or knockdown of GSK3βpromotes autophagy to reduce cadmium‐induced apoptosis.^54^ In the study we found that SB‐216763 suppresses cardiovascular and renal inflammation and activates autophagy in cardiac and renal tissues. More important, inhibition of autophagy increases perivascular fibrosis and tubulointerstitial injury in the SB216763‐treated group. These results demonstrate that SB‐216763 protects against Aldo‐induced cardiac and renal injury by activating autophagy. As shown in Figures 4 and 5, Aldo treatment can slightly increase the protein levels of LC3‐II in the cardiorenal tissues as well as p62 degradation, indicating Aldo slightly activates autophagy. The results suggest that autophagy might be a protective mechanism following occurrence of cardiorenal injury. Unfortunately, the current level of autophagy activation is not competent to resist cardiorenal injury induced by Aldo. Therefore, additional autophagy activation induced by SB‐216763 plays an important role in inhibiting Aldo‐induced cardiorenal injury.

## 5 CONCLUSION

The current study demonstrated that a GSK‐3β inhibitor, SB‐216763, suppresses aldosterone‐induced cardiac, and renal injury in by increasing autophagy activation, thus offering a new target for prevention of cardiac and renal injury.

## CONFLICTS OF INTEREST

The authors report no conflict of interest.

## AUTHORS’ CONTRIBUTION

Planned experiments: X‐LC; Performed experiments: Y‐DZ, X‐JD, H‐YD, W‐SP, and N‐FG; Analyzed data: Q‐LZ; Contributed reagents or other essential material: Y‐DZ and W‐SP; Wrote the paper: X‐LC.
